# Dependence of Neuroprosthetic Stimulation on the Sensory Modality of the Trigeminal Neurons Following Nerve Injury. Implications in the Design of Future Sensory Neuroprostheses for Correct Perception and Modulation of Neuropathic Pain

**DOI:** 10.3389/fnins.2019.00389

**Published:** 2019-05-01

**Authors:** Assunta Virtuoso, Celia Herrera-Rincon, Michele Papa, Fivos Panetsos

**Affiliations:** ^1^Division of Human Anatomy – Neuronal Networks Morphology Lab, Department of Mental, Physical Health and Preventive Medicine, University of Campania “Luigi Vanvitelli”, Naples, Italy; ^2^Neuro-computing & Neuro-robotics Research Group, Universidad Complutense de Madrid, Madrid, Spain; ^3^Instituto de Investigación Sanitaria San Carlos, Hospital San Carlos de Madrid (IdISSC), Madrid, Spain; ^4^Silk Biomed, Madrid, Spain

**Keywords:** amputation, neuroprosthesis, prostheses, ganglia, primary sensory neurons, Trk, electrical stimulation, neurodegeneration

## Abstract

Amputation of a sensory peripheral nerve induces severe anatomical and functional changes along the afferent pathway as well as perception alterations and neuropathic pain. In previous studies we showed that electrical stimulation applied to a transected infraorbital nerve protects the somatosensory cortex from the above-mentioned sensory deprivation-related changes. In the present study we focus on the initial tract of the somatosensory pathway and we investigate the way weak electrical stimulation modulates the neuroprotective-neuroregenerative and functional processes of trigeminal ganglia primary sensory neurons by studying the expression of neurotrophins (NTFs) and Glia-Derived Neurotrophic Factors (GDNFs) receptors. Neurostimulation was applied to the proximal stump of a transected left infraorbitary nerve using a neuroprosthetic micro-device 12 h/day for 4 weeks in freely behaving rats. Neurons were studied by *in situ* hybridization and immunohistochemistry against RET (proto-oncogene tyrosine kinase “rearranged during transfection”), tropomyosin-related kinases (TrkA, TrkB, TrkC) receptors and IB4 (Isolectin B4 from Griffonia simplicifolia). Intra-group (left vs. right ganglia) and inter-group comparisons (between Control, Axotomization and Stimulation-after-axotomization groups) were performed using the mean percentage change of the number of positive cells per section [100^∗^(left–right)/right)]. Intra-group differences were studied by paired *t*-tests. For inter-group comparisons ANOVA test followed by *post hoc* LSD test (when *P* < 0.05) were used. Significance level (α) was set to 0.05 in all cases. Results showed that (i) neurostimulation has heterogeneous effects on primary nociceptive and mechanoceptive/proprioceptive neurons; (ii) neurostimulation affects RET-expressing small and large neurons which include thermo-nociceptors and mechanoceptors, as well as on the IB4- and TrkB-positive populations, which mainly correspond to non-peptidergic thermo-nociceptive cells and mechanoceptors respectively. Our results suggest (i) electrical stimulation differentially affects modality-specific primary sensory neurons (ii) artificial input mainly acts on specific nociceptive and mechanoceptive neurons (iii) neuroprosthetic stimulation could be used to modulate peripheral nerve injuries-induced neuropathic pain. These could have important functional implications in both, the design of effective clinical neurostimulation-based protocols and the development of neuroprosthetic devices, controlling primary sensory neurons through selective neurostimulation.

## Introduction

Peripheral nerve injuries directly affect primary sensory neurons inducing structural and functional alterations to the cell bodies of the damaged axons. The rough endoplasmic reticulum of the primary sensory neurons undergoes a structural reorganization, known as chromatolysis, in which cell volume increases, the nucleus is displaced to the periphery of the cell and Nissl bodies get disorganized. Neurons switch from “transmitting” to “repairing/growing” functional mode. At this stage cellular metabolism is mainly committed to the repair of the damaged structures, promoting axonal regeneration through the expression of growth-associated proteins, tubulin, actin, neuropeptides, and cytokines (Fu and Gordon, 1997; Boyd and Gordon, 2003). Phenotypic changes are preceded by an immediate expression of early genes and transcription factors, probably induced via an injury-dependent activation of different signal transduction mechanisms (Abe and Cavalli, 2008; Tedeschi, 2012). Primary sensory neurons are located in the trigeminal (TG) and dorsal root ganglia (DRG). Although phenotypic integrity of adult ganglion neurons is determined by both, anterograde, and retrograde communication with their target tissues (Hamburger and Levi-Montalcini, 1949; Delcroix et al., 2003; Hippenmeyer et al., 2004) regeneration and repair processes are probably triggered only by retrograde communication signals from the injured periphery (Lundborg, 2005).

TG-DRG sensory neurons express NTF’s (neurotrophins) [nerve growth factor (NGF); brain-derived neurotrophic factor (BDNF); neurotrophin 3 (NT3); Neurotrophin 4/5 (NT-4/5)] and GDNF’s family ligands [glial derived neurotrophic factor (GDNF); neurturin (NRTN); artemin (ARTN); persephin (PSPN)], as well as their receptors from development to adult age: tropomyosin-related kinases TrkA for NGF, TrkB for BDNF-NT4/5, TrkC for NT3, low affinity p75 neurotrophin receptor for NTF ligands; and RET (proto-oncogene tyrosine kinase “rearranged during transfection”) for GDNF ligands (Davies, 1997; Airaksinen and Saarma, 2002). Both families are responsible for the survival of sensory and motor ganglia neurons during development (Mu et al., 1993; Davies, 1997; Fundin et al., 1997), for the maintenance of the phenotypic integrity during maturity (Lindsay and Harmar, 1989; Lewin and Barde, 1996) and for the regeneration and repair processes in case of injury (Munson et al., 1997; Airaksinen and Saarma, 2002; Boyd and Gordon, 2003).

**FIGURE 1:**
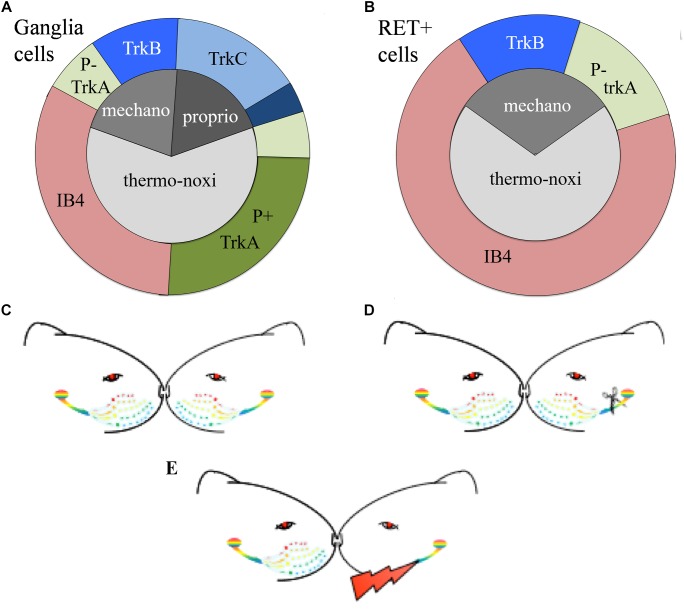
**(A,B)** Schematic representation of the distributions of TG neurons according to the expression of their receptors. **(A)** Approximately 65% of trigeminal neurons are thermo-nociceptors; half of them express TrkA and the rest are IB4 positive. Mechanoreceptors/proprioceptors express either TrkB or TrkC and some of them both; non-specific mechanoreceptors/proprioceptors are represented by deep blue. **(B)** RET-positive ganglia neurons co-express IB4, trkA, or trkB or none of them. RET-positive neurons do not cover all trkA- and trkB-positive cells. Non-frequent or controversial co-expressions like TrkB-TrkC are not depicted in this figure. **(C–E)** Schematic representation of the experimental paradigm. **(C)** Control “C” animals with bilateral intact trigeminal nerves and ganglia; **(D)** axotomized “A” animals with unilateral-left side axotomized trigeminal neurons and **(E)** stimulated “S” animals with electrode implant and electrical stimulation of the axotomized cells.

The expression of the neurotrophic factors in both, TG and DRG, is modality-specific and can be used as a biomarker to characterize the different types of primary sensory neurons (Wright and Snider, 1995; Genç et al., 2005; Liu and Ma, 2011). Roughly, TrkA characterizes small nociceptive neurons, TrkB cutaneous mechanoceptive and TrkC proprioceptive neurons although some neuronal subtypes co-express multiple neurotrophic factors (Fünfschilling et al., 2004; Hsieh et al., 2018). Mechanoceptors and proprioceptors are large diameter cells giving origin to large diameter myelinated axons. They mainly respond to low threshold mechanical stimuli and express the TrkB receptor for BDNF or NT4/5 and the TrkC receptor for NT3 respectively. Thermo-nociceptors are small diameter cells with thin unmyelinated axons mainly responding to noxious and thermal stimuli. Approximately half of the thermo-nociceptors synthesize neuropeptides and express the TrkA receptor (Averill et al., 1995) while the other half possess IB4-positive surface glyco-conjugates that bind the lectin Isolectin B4 (IB4) from Griffonia simplicifolia (Silverman and Kruger, 1990) (Figure 1A).

Easier to handle is an alternative classification of TG sensory neurons based on the expression of RET-receptor for GDNF. RET is expressed with variable percentages in all ganglia, spanning from 25% in human TG to 60% in adult mouse DRG (Luo et al., 2009; Flowerdew et al., 2013). RET-positive population is formed by the large mechanoceptive but non-proprioceptive neurons and the small diameter non-peptidergic thermo-nociceptive cells. The former, also called “early” RET neurons, encompass approximately half of the large diameter DRG population. They partially co-express TrkB but they don’t co-express TrkA or TrkC, suggesting they are mechanoreceptors and not nociceptors nor proprioceptors. The latter constitute the majority of RET-positive cells. They co-express IB4 and emerge from neurons expressing TrkA during the late embryonic stages (Molliver et al., 1997; Kramer et al., 2006; Bourane et al., 2009; Luo et al., 2009) (Figure 1B).

Peripheral nerve injuries radically change the expression of both, neurotrophins and receptors (Funakoshi et al., 1993; Wheeler et al., 1998; Bergman et al., 1999; Terenghi, 1999; Lee et al., 2001; Richner et al., 2014; Sanna et al., 2017). Additionally retrogradely transported NGF activates the expression of genes responsible for neural repair and survival (Skaper, 2008).

In previous works we showed that chronic neuroprosthetic stimulation of amputated peripheral nerves preserves the somatosensory cortex from the physiological, anatomical, and functional degenerative processes originated by the deafferentation (Herrera-Rincon et al., 2012). Furthermore we showed that neuroprosthetic stimulation maintains the functional properties of the cortical tissue close to “normality.” These central effects suggest that electrical stimulation of the injured nerve preserves some “transmitting” functions in the axotomized peripheral neurons although peripheral injury has switched them to the “repair” regime.

In the present study we focused on the initial part of the somatosensory pathway and we proposed to identify the way weak electrical stimulation modulates the neuroprotective-neuroregenerative and functional processes of TG primary sensory neurons. We hypothesized that orthodromic spikes generated by the artificial stimulation induce a partial gene expression/cell signaling to the neurons that may be responsible for the prevention of some of the injury-induced changes in the axotomized cells. To test our hypotheses we investigated the expression of neurotrophic factors’ binding receptors of TG primary sensory neurons following infraorbital nerve axotomy and electrical stimulation and we compared it with the expression in TG cells after infraorbital nerve axotomy and non-manipulated infraorbital nerve.

## Materials and Methods

### Animal Care

Eighteen female adult Wistar rats (220–250 g body weight) were used for the experiments. All surgeries were performed under general anesthesia (i.p. injection of a mixture of ketamine 80 mg/kg, and xylazine 20 mg/kg) in aseptic conditions. The body temperature was kept constant through the aid of a thermostat-controlled heating lamp (Ceramic Heat Emitter with Remote Sensor Thermostat 500R. Sun Coast Sugar Gliders^®^). The animals status was observed carefully, taking care of any abnormal or absent reflex. A mix of analgesic (Buprenorphine, 0.01–0.05 mg/kg, i.m., Buprex^®^), non-steroidal anti-inflammatory (Meloxicam, 2.0 mg/kg, s.c., Metacam^®^) and antibiotic (Enrofloxacin, 1.0 mg/kg, s.c.) drug was administered after surgery for 3 days.

Animals were allowed free moving in transparent methacrylate cages to socially interact among them, had free access to food and water and followed a 12 h/12 h day/night cycle in the post-operative period. Animal handling, housing, surgery, and sacrifice were approved by the animal care committee and carried out according to the current Spanish national legislation (R.D. 223/88) and EU directives on this matter (86/609/EC).

### Experimental Procedure

To study the effect of electrical stimulation on the axotomized TG we used the experimental model of irreversible transection of the infraorbitary nerve associated to a neuroprosthetic micro-device for stimulation of the injured axons described in Herrera-Rincon et al. (2012) and Herrera-Rincon and Panetsos (2014) (Figure 1C–E). After left infraorbitary nerve transection the proximal nerve stump was inserted into a neuroprosthetic micro-device containing the stimulation electrodes.

### Neuroprosthetic Stimulation

**FIGURE 2:**
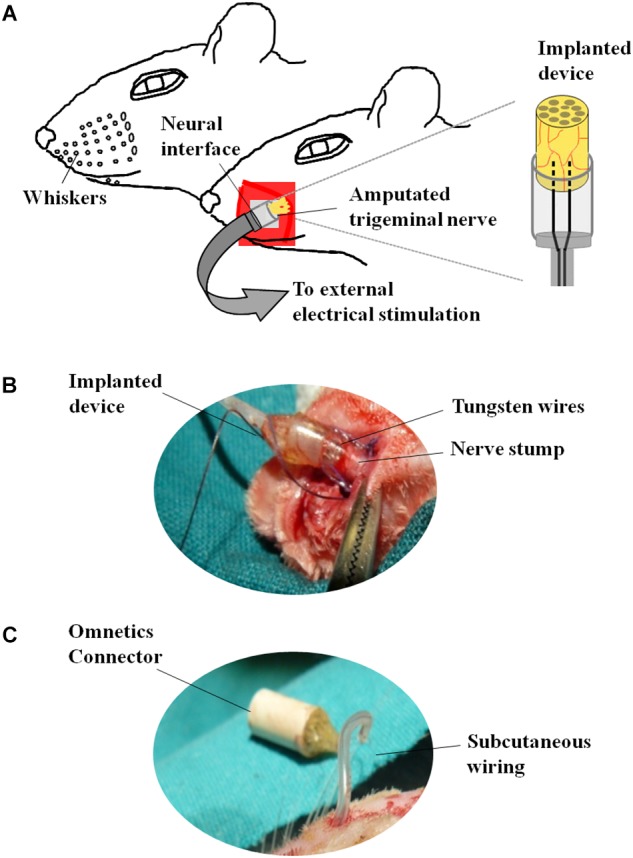
Neuroprosthetic stimulation device. **(A)** Schematic representation of the implant. **(B)** After fixing the screws to the skull, the infraorbital nerve is dissected taking all the fascicles that innervate the vibrissae; immediately after cutting, the distal end of the nerve is inserted into the implant and placed in contact with the electrodes; the epineurium is then sutured to the silicone implant. **(C)** Before the nerve section, the implant is subcutaneously tunneled to connect the skull to the snout.

The neuroprosthetic stimulator was composed of two stimulation electrodes placed at the same level, at a distance of 0.5 mm between each other and connected to a circular connector (Omnetics^®^) fixed in the cranium of the animal (Figure 2). The electrodes consisted of two tungsten wires (8.0 cm length, Ø = 50 mm), coated with Teflon^®^PFA but in the tip. The proximal part of the wires was integrated into a tubular silicone guide (2.0 mm internal diameter and 2.5 cm length, Figure 2A,B). The device was open from the side of the tips of the electrodes to allow the insertion of the left ION stump following complete nerve transection (Figure 2B). A 0.6 mm internal diameter vinyl tube was glued to the silicon guide and the distal part of the wires was inserted into it (Figure 2B). Vinyl tube and wires were directed subcutaneously to the skull of the animal and welded to a female circular connector (Omnetics^®^) attached to the scalp (Figure 2C) through an anchoring system consisting of four microsurgery screws, and covered with dental cement. The device was externalized through the scalp and connected to an external generator of voltage pulses (Cygnus-PG4000, Delaware Water Gap, Monroe County, PA, United States). Stimulation started immediately after surgery (S, stimulation-after-axotomization group, *n* = 6) and was applied for 4 weeks: 12 h per day, square pulses of 100 μs, 3.0 V, at 20 Hz. As an experimental control, we used axotomized animals subject to surgical implant without applying electrical stimuli (A, axotomization group, *n* = 6). As normal control we used naive, non-operated animals (C, control group, *n* = 6). Animals were deeply anesthetized and sacrificed after 4 weeks; the left (ipsilateral to the lesion, affected) and the right TG (contralateral to the lesion, non-affected) from each animal were collected, sectioned in a cryostat and processed for *in situ* hybridization and immunohistochemistry.

### “*In situ*” Hybridization and Immunohistochemistry

Amplification of the TrkA gene by RT-PCR and DIG-labeled antisense riboprobe (ribonucleic acid) synthesis were performed as described in Ventéo et al. (2012). Total RNA was isolated from wild-type adult rat TG and reverse-transcripted (RT-PCR). For TrkA, cDNA sequences were PCR amplified using the s-TGGCAGTTCTCTTTCCCCTA and as-AAAGCTC-CACACATCGCTCT primers. Amplified fragments were ligated into the pGEM-T easy vector using the TA cloning kit (Promega). Probes for TrkB, TrkC, and Ret were kindly provided by Dr. E. Castren, Dr. F. Lamballe, and Dr. V. Pachnis, respectively. Digoxigenin (DIG)-labeled antisense RNA probes were synthesized using the DIG-labeling kit (Roche), according to the manufacturer’s instructions.

Trigeminal ganglia were dissected in phosphate-buffered saline (PBS) pH 7.4 and fixed in 4% paraformaldehyde (PFA) for 1 h at room temperature, cryoprotected overnight at 4°C in 25% sucrose in PBS before embedding in OCT compound (Optimal Cutting Temperature, Tissue-Tek). 14 μm sections were cut on a cryostat and serially collected on ProbeOn Plus microscope slides (Fisher Scientific).

*In situ* hybridization was performed according the procedure used by Bourane et al. (2007). TG sections were incubated with DIG-labeled antisense riboprobes at 65° overnight, followed by two washes in 1 × SSC (saline-sodium citrate), 50% formamide, and 0.1% Tween-20 at 65°C for 30 min; after the blocking (2% blocking reagent and 20% inactivated sheep serum) the slides were incubated with anti-DIG-alkaline-phosphatase (AP)-conjugated antibody (Roche Diagnostics), washed and revealed using NBT/BCIP staining. For IB4 immunohistochemistry, cryostat sections were blocked into 1% BSA (Bovine Serum Albumin) + 0.1% Triton in PBS for 1 h and stained for IB4-Biotin (10 mg/ml, Sigma) followed by ExtrAvidin-FITC conjugated (fluorescein isothiocyanate, 1/400, Sigma).

### Cell Counting and Distributions

Slides were imaged with a Zeiss Axioskope 2 light microscope equipped with high-resolution digital camera (C4742-95, Hamamatsu Photonics, Italy). Measurements of markers were accomplished using computer assisted image analysis system [MCID 7.1; Imaging Res., Inc., Canada as previously reported in Cirillo et al. (2011)]. TrkA-, TrkB-, TrkC-, IB4-, and RET-positive sensory neurons were identified and counted on sequential slides of left (ipsilateral/affected) and right (contralateral/non-affected) TG of control, axotomized and stimulated animals under 10x objective. Tissue sections from left and right TG were divided in six histological series; five of them were used to label for the above-mentioned markers.

**FIGURE 3:**
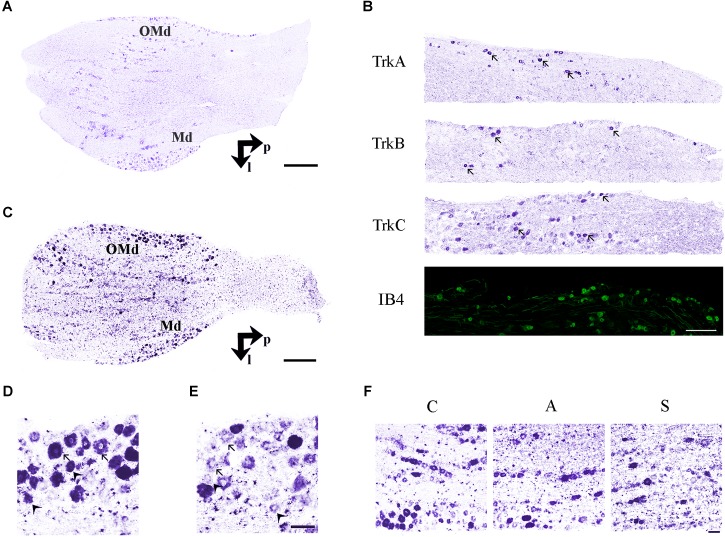
**(A)** *In situ* hybridization using TrkB probe showing the histological architecture in a control TG. OMd, ophthalmic-maxillary division; Md, mandibular division; p, posterior; l, lateral. Scale bar = 100 μm. **(B)** Panoramic microphotographs showing the distribution of neuronal subpopulations as defined by the expression of TrkA, TrkB, TrkC, and IB4 in a control TG. Arrows indicate positive neurons. Scale bar = 100 μm. **(C)** *In situ* hybridization of RET proto-oncogene mRNA. OMd, ophthalmic-maxillary division; Md, mandibular division; p, posterior; l, lateral. Scale bar = 100 μm. **(D)** Microphotographs for RET-positive neurons. Large diameter cells, Ø ≥ 20 μm, were classified as putatively mechanoceptive or proprioceptive neurons (arrow) and small diameter ones, 8 μm ≤ Ø < 20 μm, as putatively thermo-nociceptive neurons (arrowhead). **(E)** Small and large RET-negative cells were also present in all RET-reacted slides. Scale bar = 50 μm for both images. **(F)** Representative microphotographs of RET-positive neurons in the ophthalmic-maxillary division from C, A, and S animals. Scale bar = 50 μm.

Two different systematic random samplings (1/3 ratio) were performed for RET-labeled sections, the former in the whole ganglion and the latter in the ophthalmic-maxillary (OM) region, which includes the neuronal bodies directly involved into the axotomization and the neurons having projections in the ophthalmic nerve. It is important to underlie that even in this case, the sampling is not restricted to the maxillary neurons because, in rodents, the maxillary division is not anatomically distinguishable from the ophthalmic one. The ophthalmic-maxillary division appears like a cephalic-median area, blocked in by parallel lines and occupying about two-thirds or more of the ganglion (Allen, 1924), while the mandibular one is located postero-laterally and it is characterized by cells clustered in a lateral protuberance (Shellhammer, 1980) (Figure 3A).

We counted the totality of positive cells with neural morphology establishing target parameters like maximum diameter, area and form factor. The maximum diameter was considered to be the maximum internal distance perpendicular to the curved chord; the area was calculated by counting pixels inside the outline borders of the targets; the form factor is a standard estimate of circularity that relates perimeter to area. In all cases cell nuclei were used as counting units and the mean number of positive neurons per section for each ganglion and marker was calculated. In the case of the RET receptor we also counted the RET-negative neurons in both regions of interest. Maintaining the measurements criteria, RET-negative neurons were detected by inverting the hue-intensity parameters. Measurements were performed by a single-blinded investigator.

Neurons were classified as large diameter putatively mechanoceptors/proprioceptors (Ø > 20 μm) and small diameter putatively thermo-nociceptors (8 μm ≤ Ø < 20 μm). Lower diameter cells were identified as satellite cells and were not considered for counting (Ø < 8 μm).

### Experimental Design and Statistical Analysis

The experimental groups (C, A, S) have been determined by the need to compare electrically stimulated ganglia with sectioned but not stimulated ones as well as with ganglia from non-manipulated animals. Sample size was determined by means of a non-central F distribution adjusted for an 80% test power, for a maximum difference of the means less or equal twice the standard deviation of the variables. Calculi were performed with the aid of Statgraphics Centurion XVII© software (Kilkenny et al., 2009).

Intra-group (left vs. right ganglia) and inter-group (between C, A, and S groups) comparisons of TrkA-, TrkB-, TrkC-, IB4-, and RET-expression were performed using the mean percentage change of the number of positive cells per section [100^∗^(left–right)/right)] or Δ%. Intra-group differences were studied by paired *t*-tests. For inter-group comparisons ANOVA test followed by *post hoc* LSD test (when *P* < 0.05) were used. Significance level (α) was set to 0.05 in all cases. Statistical values are reported as mean ± standard error of the mean (SEM).

## Results

Trigeminal neurons typically displayed large, centrally-placed nuclei (Shellhammer, 1980). TrkA-, TrkB, TrkC-, IB4- (Figure 3B) and RET-positive sensory neurons (Figure 3C) were identified and counted on the slides of left (ipsilateral/affected) and right (contralateral/non-affected) TG. They were distributed in a rather uniform pattern throughout the ophthalmic, maxillary, and mandibular TG division (Figure 3C). Control, amputated, and stimulated TG neurons displayed left–right asymmetries in both, the number and dimensions of the cells. Asymmetries were found in both, the OM and the whole TG, as expected by the bibliography (LaMendola and Bever, 1997; Koltzenburg et al., 1999; Lagares and Avendaño, 2000).

### RET-Expressing Neurons

In the OM, intra-group analysis showed a statistically significant decrease of the RET^+^ neurons following amputation of the peripheral nerve becoming even more prominent after electrical stimulation of the proximal stump: the slightly positive Δ% of RET^+^ neurons in favor of the left TG (10.2 ± 0.7 left vs. 9.9 ± 0.5 right, Δ% = +2.1%) under control conditions became significantly negative after the peripheral deafferentation (8.3 ± 1 left vs. 10.9 ± 0.8 right,Δ% = −24.7%) and reached Δ% = −37.8% (6.2 ± 0.2 left vs. 10 ± 0.7 right) under electrical stimulation of the transected nerves (*P* < 0.05 in both cases). These changes were also reflected in the inter-group differences (*P* < 0.05 C-A; *P* < 0.01C-S). Details are shown in Table 1.

**Table 1:** OM region.

	Intra-group comparisons		Inter-group comparisons	
Groups	Δ%	*P*-value	ANOVA	0.003^∗∗^
C	2.1 ± 3.8	0.604	C–A	0.012^∗^
A	−24.7 ± 5.2	0.012^∗^	C–S	0.004^∗∗^
S	−37.8 ± 5.6	0.042^∗^	A–S	0.154

*Intra-and inter-group Δ% comparisons of mean numbers of RET-positive neurons per slide.*

To better understand the behavior of the OM neurons in the different experimental conditions, we distinguished between small and large RET^+^ cells, the former being putative thermo-nociceptors and the latter mechanoceptors/proprioceptors (Luo et al., 2009; Hsieh et al., 2018), classified according to the maximum diameter of their somata (8 μm ≤ Ø < 20 μm for the former, Ø ≥ 20 μm for the latter, Figure 3D,E).

The equilibrium between left and right OM RET^+^ neurons in control animals was the result of the opposite left–right ratios of small and large neurons in this area: a higher number of small RET^+^ and a lower number of large RET^+^ neurons in the left TG (5.8 ± 1.2 left vs. 4.9 ± 0.8 right, Δ% = +27.5% for small neurons; 4.3 ± 1.8 left vs. 5.0 ± 1 right, Δ% = −14.1% for large neurons). Axotomy provoked a drastic drop in the population of the small neurons that reached Δ% = −35.6% (4.7 ± 1 left vs. 7.4 ± 1 right), while the population of large diameter neurons increased up to Δ% = +21.9% (3.5 ± 0.4 left vs. 3.5 ± 0.7 right). Neuroprosthetic stimulation attenuated the drop of the small neurons (4.8 ± 0.2 left vs. 5.4 ± 0.6 right, Δ% = −7.4%) while it decreased the number of large diameter cells, whose Δ% fell to −69.3% (1.3 ± 0.2 left vs. 4.6 ± 0.6 right) (*P* < 0.05). Details are shown in Table 2. Experimental manipulations did not affect the morphology of the cells (Figure 3F).

**Table 2:** OM region.

	Small+		Large+	
Groups	Δ%	*P*-value	Δ%	*P*-value
C	27.5 ± 42.4	0.638	−14.1 ± 25	0.697
A	−35.6 ± 11.9	0.082	21.9 ± 38	0.985
S	−7.4 ± 13.4	0.523	−69.3 ± 7.3	0.049^∗^

*Intra-group Δ% comparisons of mean numbers of RET-positive neurons per slide, of small (Ø ≤ 20 μm) and large (Ø > 20 μm) neural cells.*

**FIGURE 4:**
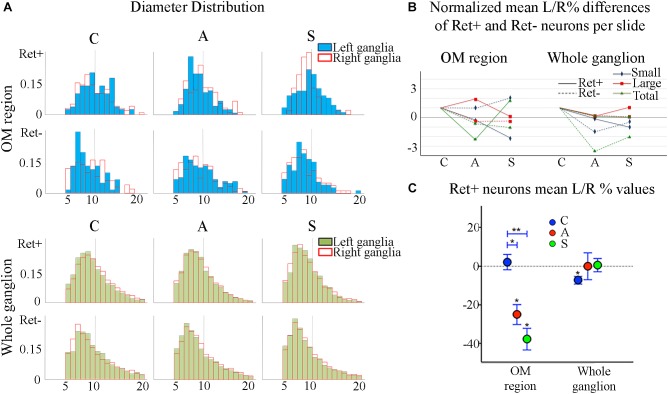
**(A)** Normalized histograms displaying diameter distribution of RET-positive and RET-negative neurons of representative C, A, and S animals. Blue histograms correspond to neurons of the ophthalmic-maxillary (OM) area, green to the whole ganglion. Top line display RET-positive and bottom line RET-negative neurons in both cases. Black perimeter filled bars correspond to right/non-manipulated ganglia; red perimeter empty bars correspond to the left/manipulated ganglion. Dashed vertical line indicates the border between small to large diameter neurons (Ø = 20 μm). Bin amplitude 2 μm. Changes in left–right distributions passing from C to A and S animals reflect the effects of the deafferentation and of electrical stimulation described in the text. **(B)** Graphic representation of the normalized mean left–right percentage differences (L/R%) in the number of RET-positive and RET-negative neurons in C, A, and S animals. L/R% are shown separately for small, large and the totality of the neurons in the ophthalmic-maxillary division (OM, left) and of the whole ganglia (right). Normalization was performed by dividing C, A, and S values by C value. **(C)** Comparison of L/R% for RET-positive neurons in the ophthalmic-maxillary region and the whole ganglion. Left: in OM, control TG neurons display a slightly positive L/R% (blue circles), which is transformed to a significant negative L/R% after axotomization (red circles, paired *t*-test, *P* < 0.05) and it becomes even stronger under electrical stimulation of the transected nerve (green circles). Right: in the whole ganglion, RET-positive neurons show a significant negative L/R% in control animals that is abolished following axotomization and axotomization+electrical stimulation of the stump (red and green circles respectively).

The reduction of the number of the small RET^+^ neurons in the left -axotomized- OM could be due either to the RET downregulation or to the increase of the volume of these neurons (Gersh and Bodian, 1943; Gutmann, 1964; Deitch and Rubel, 1989) making them exceed the limit of Ø = 20 μm and get counted as large RET^+^ ones. RET downregulation of the small cells can be excluded because axotomization did not change the number of the small RET-negative neurons (Δ% = 45.5 in C group; Δ% = 45.4 in A group; Δ% = 44.1 in S group). Intra-group analysis provided evidence in favor of the increase of the volume (Δ% = −3.7 C; Δ% = 1,8 in A group; Δ% = 7,6 in S group). Details are shown in Table 3. These data were further supported by the distribution histograms of the diameters of the OM small cells in C, A, and S animals (Figure 4A,B-left).

**Table 3:** OM region (left) and whole trigeminal ganglion (right).

	OM region				Whole trigeminal ganglion			
	Small+		Large+		Small+		Large+	
Groups	Δ%	*P*-value	Δ%	*P*-value	Δ%	*P*-value	Δ%	*P*-value
C	−3.7 ± 3.0	0.333	2.6 ± 1.6	0.241	1.4 ± 0.1	0.007^∗∗^	1.2 ± 0.2	0.035^∗^
A	1.8 ± 2.0	0.435	4.3 ± 3.0	0.244	−0.3 ± 1.4	0.838	0.0 ± 1.3	0.975
S	7.6 ± 3.0	0.109	−0.1 ± 6.1	0.991	−1.4 ± 0.4	0.077	1.3 ± 0.8	0.224

*Intra-group Δ% comparisons of mean maximum diameters of RET-positive neurons per slide of small (Ø ≤ 20 μm) and large (Ø > 20 μm) neural cells.*

With respect to the large-size RET^+^ OM neurons, the increase of their number in axotomized TG could be explained by a RET upregulation induced by the experimental manipulation. Such upregulation was confirmed by the decrease of the number of large RET-negative cells in left-axotomized-TG (4.4 ± 1.1 left vs. 2.4 ± 0.5 right, Δ% = 113.3 for C group; 4.2 ± 0.1 left vs. 3.5 ± 0.7 right, Δ% = 44.9 for A group). The same argument is valid for the effect of the neuroprosthetic stimulation: volume increase of the large RET^+^ neurons was clearly reversed by the neuroprosthetic stimulation (Δ% = 2.6 in C group; Δ% = 4.3 in A group; Δ% = −0.1 in S group). Probably, the same occurred in the small neurons, but this effect was masked by the noticeable reverse of the large neurons that reduced their diameters to under 20 μm (Figure 4A). Details are shown in Table 3. In OM, axotomization exerted a 63.0% change to small RET^+^ neurons (Δ% = 27.5 ± 42.4 in C group; Δ% = −35.6 ± 11.9 in A group) and a 35.0% change to the large cells (Δ%=-14.1 ± 25 in C group; 21.9 ± 38 in A group).

In the whole ganglia, C animals showed a statistically significant asymmetry of RET^+^ neurons in favor of the right side (*P* < 0.05, Figure 4C). Such asymmetry was abolished by peripheral axotomy and electrical stimulation of the transected nerve (C, 163.1 ± 8.5 left vs. 176.3 ± 6.1 right, Δ% = −7.6; A, 144.9 ± 11 left vs. 146.7 ± 13 right, Δ% = −0,1; S, 133.8 ± 18 left vs. 132.7 ± 16 right, Δ% = 0,5). This behavior was mirrored in the RET^+^ sub-populations (small neurons). Moreover, the RET-negative population in the whole ganglion appeared to have an inverse tendency comparing to the RET^+^ (Figure 4B-right, C). Details are shown in Table 4.

**Table 4:** Whole trigeminal ganglion.

	Total+		Small+		Large+	
Groups	Δ%	*P*-value	Δ%	*P*-value	Δ%	*P*-value
C	−7.6 ± 1.6	0.032^∗^	−17.5 ± 3.6	0.054	11.3 ± 9.8	0.406
A	−0.1 ± 7.2	0.875	−1.5 ± 6.5	0.723	3.1 ± 8.8	0.871
S	0.5 ± 3.2	0.797	0.7 ± 1.9	0.801	0.0 ± 8.9	0.873

*Intra-group Δ% comparisons of mean numbers of RET-positive neurons per slide. Data are shown for the total neural population as well as for the small (Ø ≤ 20 μm) and large (Ø > 20 μm) neural cells separately.*

### TrkB – TrkC Expressing Neurons

**FIGURE 5:**
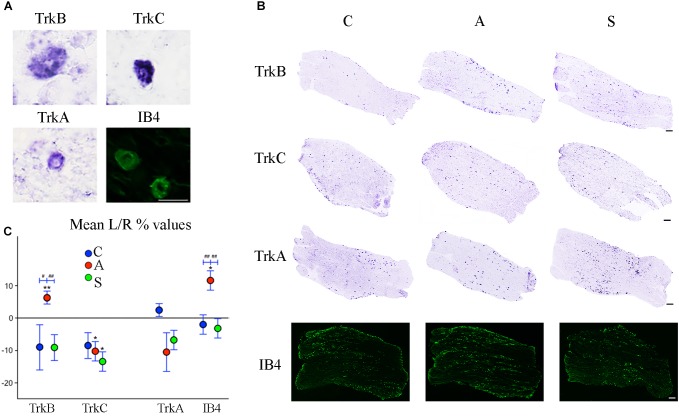
**(A)** Microphotographs of Trk- ad IB4- positive neurons from left ganglia of an axotomized animal. Scale bar = 20 μm. **(B)** Representative mechano-proprioceptors (TrkB, TrkC) and thermo-nociceptors (TrkA, IB4)-reacted slices showing positive neurons in the left ganglion of Control (C), Amputated (A), and Stimulated (S) animals. Scale bar = 50 μm for all the images. **(C)** Graphic representation of mean percentage changes in the number of Trk- and IB4-positive neurons between left and right TG indicated as L/R (%). Intra-group comparisons for mechano/proprioceptors show a significant increase in the number of TrkB-positive neurons and a decrease in the number of TrkC-positive ones after axotomization. Electrical stimulation of the transected nerve prevents such effect in the case of TrkB neurons while it potentiates the decrease of TrkC-positive cells. A similar effect is observed in the case of thermo-nociceptors: the number of IB4-positive increases while the number of TrkA-positive neurons diminishes, although TrkA decrease does not reach significant levels. Electrical stimulation of the amputated nerve reverses axotomy effects or prevents changes for both markers. Inter-group comparisons show significant differences between A and C-S groups in both TrkB- and IB4-positive neurons. Intragroup significant differences are indicated by asterisks (paired *t*-test, ^∗^*P* < 0.05; ^∗∗^*P* < 0.01). Intergroup significant differences are indicated by hashtags (one-way ANOVA followed by *post hoc* LSD test, #*P* < 0.05; ##*P* < 0.01).

TrkB and TrkC mostly label > 20 μm diameter neurons (Figure 5A), considered to correspond mainly to mechanoceptors/proprioceptors.

Both intra- and inter-group assessment of the whole slide area revealed a very significant increase in the number of TrkB-positive neurons in the axotomized/left ganglia. Left–right percentage difference passed from Δ% = −9 (44 ± 2 left vs. 48 ± 2 right) in C group to Δ% = 6 (50 ± 3 left vs. 47 ± 4 right) in A group (*P* < 0.01 for both, A left–right intra-group and C-A inter-group comparisons; Figure 5B,C). Electrical stimulation of the transected nerve provided protection to the trigeminal neurons following nerve injury (Δ% = −9; *P* < 0.01 A-S; Figure 5B,C). Details are shown in Tables 5, 6.

**Table 5:** Whole trigeminal ganglion.

	Trk-A		Trk-B		Trk-C		IB4	
				
Groups	Δ%	*P*-value	Δ%	*P*-value	Δ%	*P*-value	Δ%	*P*-value
C	2.30 ± 1.92	0.362	−8.91 ± 6.62	0.241	−8.49 ± 4.24	0.086	−2.00 ± 2.86	0.533
A	−10.51 ± 5.61	0.158	6.31 ± 1.83	0.009^∗∗^	−10.11 ± 3.19	0.022^∗^	11.50 ± 2.68	0.028^∗^
S	−6.97 ± 2.39	0.095	−9.07 ± 3.87	0.103	−13.37 ± 2.99	0.010^∗^	−3.11 ± 3.33	0.408

*Intra-group Δ% comparisons of mean numbers of TrkA-, TrkB-, TrkC-, and IB4-positive neurons per slide.*

**Table 6:** Whole trigeminal ganglion.

Comparisons	Trk-A	Trk-B	Trk-C	IB4
ANOVA	0.191	0.036^∗^	0.619	0.008^∗∗^
C–A	–	0.043^∗^	–	0.009^∗∗^
C–S	–	0.984	–	0.805
A–S	–	0.006^∗∗^	–	0.009^∗∗^

*Inter-group Δ% comparisons of mean numbers of TrkA-, TrkB-, TrkC-, and IB4-positive neurons per slide. Trigeminal ganglion Ophthalmic-Maxillary (OM) region and whole trigeminal ganglion statistical tables of intra- and inter-group comparisons of C, A, and S animals (n = 6) show mean percentage differences (Left minus Right) of mean numbers of positive neurons per slide, or mean maximum diameters per slide, indicated as Δ%. Left refers to ipsilateral/affected and right to contralateral/non-affected ganglia. In intra-group comparisons, P-values of paired t-tests are given for each group. In inter-group tables, one-way ANOVA P-value is indicated in the top. P-values for “post hoc” comparisons are indicated among C, A, and S groups, two-by-two in the cases ANOVA showed a statistically significant difference. Values represent the mean ± SEM of the distribution. Statistically significant differences are highlighted by an asterisk (P < 0.05) or two asterisks (P < 0.01).*

TrkC-expressing neurons showed a significant decrease in the axotomized animals (*P* < 0.05), this behavior becoming even more evident in the stimulation group (*P* < 0.01) with left–right percentage differences shifting from Δ% = −8 (129 ± 18 left vs. 141 ± 18 right) in the control group to Δ% = −10 (128 ± 11 left vs. 141 ± 9 right) and then to Δ% = −13 (104 ± 7 left vs. 120 ± 6 right) in axotomization and in stimulation-after-axotomization groups respectively (Figure 5B,C). Details are shown in Tables 5, 6.

### TrkA – IB4 Expressing Neurons

TrkA- and IB4-expression was detectable in small neurons (Ø < 20 μm, Figure 5A), considered to be chiefly thermo-nociceptors.

TrkA cells counting revealed a decrease in the whole slides number of positive neurons in both, the axotomized and axotomized-stimulated TG. Left–right percentage difference of neurons number declined from Δ% = 2 (105 ± 3 left vs. 103 ± 3 right) in C group to Δ% = −11 (88 ± 10 left vs. 102 ± 17 right) and Δ% = −7 (94 ± 13 left vs. 100 ± 11 right) in A and S groups respectively (Figure 5B,C). Details are shown in Tables 5, 6.

IB4-labeling uncovered a conspicuous increase of the number of neurons in the left-axotomized-ganglia where left–right percentage differences passed from Δ% = −2 (188 ± 6 left vs. 192 ± 2 right) under control conditions to Δ% = 12 (200 ± 32 left vs. 179 ± 28 right, *P* < 0.05; *P* < 0.01C-A) after transection of the peripheral nerve. Electrical stimulation of the amputated nerve reverted axotomization-induced modification bringing IB4-labeled neurons back to the C levels (184 ± 12 left vs. 190 ± 10 right, Δ% = −3; *P* < 0.01A-S; Figure 5B,C). Details are shown in Tables 5, 6.

## Discussion

In previous works we have studied the way electrical stimulation of transected peripheral nerves counteracts the neurodegenerative processes triggered in the central nervous system by the peripheral deafferentation (Herrera-Rincon et al., 2012; Herrera-Rincon and Panetsos, 2014).

Here we report for the first time the dynamics of the expression patterns of RET and Trks receptors for GDNF and NTF respectively, as well as IB4-immunoexpressing cells among primary trigeminal sensory neurons under different functional conditions: normality, after 4 weeks of full irreversible transection of the infraorbital nerve and after 4 weeks of artificial stimulation of the axotomized cells. Following nerve axotomy and electrical stimulation RET- and Trk-expression patterns indicate that sensory TG neurons express NGF, BDNF/NT4, GDNF, NT3 receptors at levels similar to those found in physiological conditions, although they had presumably switched to regeneration-repair state due to the injury (Sebert and Shooter, 1993; Tonra et al., 1998; Shen et al., 1999a; Terenghi, 1999; Lee et al., 2001; Karchewski et al., 2002; Geremia et al., 2010; Hougland et al., 2013). Probably, the coexistence of the functional and the repair states is a non-stable dynamic process.

### RET Expression

The count of RET-expressing neurons in the OM region showed a remarkable decrease in the axotomized TG and a further decrease after electrical stimulation. Small RET-expressing neurons are presumably non-peptidergic and linked to the perception of thermal-noxious stimuli; they are mostly IB4-positive (Molliver et al., 1997; Bourane et al., 2009; Luo et al., 2009). Additional evidences suggest that larger cells work as low threshold mechanoceptive neurons and mainly express TrkB (Terenghi, 1999; Kramer et al., 2006) with some possible limited overlapping (Kashiba et al., 2003; Bourane et al., 2009). In the Control group we found a prevalence of the small neurons compared to the larger ones. 4-weeks after axotomy, the small neurons decreased while the number of the large neurons increased. Neurostimulation had an opposite effect and drastically diminished the RET-expressing large cells, as an attempt to revert the axotomization response. The low power of the test due to the small size of the samples (Krzywinski and Altman, 2013) together with the high left–right variability of TG neurons (Lagares and Avendaño, 2000) did not allow to reach small *P*-values, making biological interpretation of the results difficult (Krzywinski and Altman, 2013).

The increment in the number of the large RET-positive neurons in axotomized animals is in total agreement with precise observations by Bennett et al. (2000), who found a significant increment mainly of the RET expressing large diameter cells among retrogradely-labeled axotomized DRG neurons, 2 weeks after injury. Previous studies are not conclusive, showing an increase in the percentages of total RET expression 1 day after the injury (Naveilhan et al., 1997) or no changes at all (Kashiba et al., 1998). The behavior of the large RET-positive neurons matches with TrkB expressing cells in both, axotomized and stimulated animals, according with prior works (Wright and Snider, 1995; Molliver and Snider, 1997; Molliver et al., 1997).

Diameter distribution of small and large RET-expressing neurons in the OM area (Figure 4A) as well as the increment of cell diameters observed after axotomization is in agreement with the literature (Bennett et al., 1998, 2000). The increase of cell diameters is probably due to the concentration of cell constituents in the soma as well as to an expansion of the volume occupied by proteins in the somata of the axotomized neurons (Gersh and Bodian, 1943; Gutmann, 1964). The inverse effects observed in the whole ganglia are probably induced by the immediate changes in the OM neurons and show the plastic effort of the whole ganglia system to counteract nerve manipulation. They could also explain the significant reduction of the volume of the neurons found in the whole ganglia of A animals passing from *P* < 0.01 and *P* < 0.05 to non-significance in small and large cells respectively (data not shown).

### Trk- and IB4-Expression

Trk- and IB4 expression in control animals is in agreement with previous studies regarding both, cell diameters and neurons distribution in TG ganglia (Ambalavanar and Morris, 1992; Bergman et al., 1999).

The loss of TrkA and TrkC cells in axotomized ganglia is in agreement with published data for TG and DRG 1 week after axotomy of the trigeminal and the sciatic nerve (Bergman et al., 1999) and for DRG ganglia following L5 sciatic nerve ligation (Shen et al., 1999b) where it is reported a decrease of the TrkA-expressing neurons during the 3 weeks after the deafferentation and the spontaneous recovery of TrkA mRNA level in 2 months.

The increase in the proportion of TrkB-expressing neurons following the axotomization is supported by studies using experimental models of injury. Ernfors et al. (1993) and Hammarberg et al. (2000) observed an increase of TrkB-positive neurons in DRG shortly after spinal cord ligation/crush while Bergman et al. (1999) observed a similar behavior in 7 days post-axotomy of the TG. To our knowledge, no one reported about TrkB evolution after 2–5 weeks. Both TrkB and BDNF expression was found to be upregulated after ligation/crush of the sciatic nerve (Ernfors et al., 1993; Tonra et al., 1998; Kashiba and Senba, 1999; Shen et al., 1999a; Fukuoka et al., 2001).

The number of IB4-expressing neurons has been demonstrated to diminish with peripheral nerve injury (Bennett et al., 1998; Kalmar et al., 2003). Literature differences in the expression levels of both, neurotrophic factors and binding receptors, could be due to the heterogeneity of experimental and data analysis procedures.

Following IoN axotomy and electrical stimulation, TG neurons express neurotrophic factor receptors at levels more proper to non-injured than to injured ones. It can be tenable that axotomy-induced changes can be spontaneously reversed over a longer period of time or forced to reverse in short-time by artificial neurostimulation.

The low effect of artificial stimulation on TrkA-reacted neurons is consistent with the theory that NGF is released from the targets of the sensory nerves, captured by nerve terminals and transported to the soma to bind TrkA receptors (Aloe et al., 2012). Under permanent axotomy nerve targets are eliminated, NGF is not transported to the soma and, consequently, TrkA receptors get less expressed. Electrical stimulation of the axotomized nerve does not replace the amputated target neither releases NGF by itself, explaining the low expression of TrkA also in stimulated animals. Our data reflect this condition, although we considered the TG neurons either directly or indirectly affected by the nerve manipulation.

Our results suggest a neurostimulation-induced modulation of TrkB in primary sensory neurons. They are also consistent with reference works in peripheral nerve regeneration showing that electrical stimulation starting immediately after cutting and repairing the femoral nerve has BDNF/TrkB-mediated neuro-regenerative effects originated at the cell body (Al-Majed et al., 2000a,b).

## Conclusion

Artificial stimulation has heterogeneous effect on the sensory neuronal subpopulations in the trigeminal ganglia. It mainly acts on RET-, TrkB-, and IB4-expressing neurons suggesting small non-peptidergic thermo-nociceptive, large mechanoceptive but non-proprioceptive neurons and cutaneous mechanoceptive neurons are more suitable to be tuned by artificial stimulation of an amputated nerve.

The different effect of the neurostimulation on the trigeminal neuronal subtypes could explain central phenomena we have reported in previous works, namely that electrical stimulation protects against deafferentation-dependent degeneration of the somatosensory pathway but does not protect against the interruption of the cholinergic input to the somatosensory cortex (Herrera-Rincon et al., 2010a,b, 2012; Herrera-Rincon and Panetsos, 2014). Such an effect could be due to the different action of the electrical stimulation over the myelinated (TrkB and TrkC) neurons, projecting epicritically to thalamus and cortex, and the unmyelinated neurons (TrkA and IB4), projecting protopatically to the reticular system and basal prosencephalon.

Regarding clinical implications, our results suggest that neurostimulation protocols, either for therapeutic applications in neuropathic pain or for the development of nerve-machine sensory neuroprostheses (Grill et al., 2009; Lotfi et al., 2011) should be designed considering sensory modality of target-ganglion neurons and the specific alterations they will elicit on each fiber/neuron type, both in the elements directly interested by the treatment and in the neighboring cells (Wagenaar et al., 2011; Bruns et al., 2013; Renna et al., 2017; Weber et al., 2006; Han et al., 2017).

## Author Contributions

AV analyzed data and wrote the manuscript. CH-R performed experiments and analyzed data. MP critically revised the manuscript and provided intellectual thoughts. FP designed the experiments, designed the manuscript, and wrote the manuscript. All authors read and approved the final manuscript to be published.

## Conflict of Interest Statement

The authors declare that the research was conducted in the absence of any commercial or financial relationships that could be construed as a potential conflict of interest.
